# *Nephrotoma* Meigen (Diptera, Tipulidae) from Xizang Autonomous Region, China

**DOI:** 10.3897/zookeys.973.46384

**Published:** 2020-10-05

**Authors:** Qi-Cheng Yang, Qi-Fei Liu, Zhao-Hui Pan, Xiao-Yan Liu, Ding Yang

**Affiliations:** 1 Hubei Insect Resources Utilization and Sustainable Pest Management Key Laboratory, College of Plant Science & Technology of Huazhong Agriculture University, Wuhan, 430070, Hubei, China Huazhong Agriculture University Wuhan China; 2 College of Plant Protection, Fujian Agriculture and Forestry University, Fuzhou, 350002, Fujian, China Fujian Agriculture and Forestry University Fuzhou China; 3 Institute of Plateau Ecology, Tibet Agriculture & Animal Husbandry University, Linzhi, 860000, Xizang, China Tibet Agriculture & Animal Husbandry University Linzhi China; 4 China Agricultural University, Beijing, 100193, China China Agricultural University Beijing China

**Keywords:** Crane flies, new species, taxonomy, Tibet, Tipulinae

## Abstract

Eight species of the genus *Nephrotoma* were previously known to occur in Xizang Autonomous Region. Here, three species are added to the fauna of Xizang. Among them two species, *N. beibengensis***sp. nov.** and *N. hanae***sp. nov.** are described and illustrated as new to science, and one species, *N. evittata* Alexander, 1935 is recorded from Xizang for the first time. The following four species are redescribed: *N. claviformis* Yang & Yang, 1987, *N. didyma* Yang & Yang, 1987, *N. nigrohalterata* Edwards, 1928, and *N. xizangensis* Yang & Yang, 1987. A key to the species of *Nephrotoma* from Xizang is presented.

## Introduction

The genus *Nephrotoma* Meigen is one of the largest genera in the family Tipulidae. It is distributed worldwide with 163 known taxa from the Palaearctic Region and 127 taxa from the Oriental Region. Until now, 96 taxa are known in China (Oosterbroek 2020). In recent years, Men et al. (2015, 2016, 2017) and Ren and Yang (2017) increased our knowledge of the genus *Nephrotoma* in China. This genus is characterized by the following features: body usually yellow with dark stripes at top of prescutum or nearly entirely black; Rs short, cell m_1_ sessile or shortly petiolate; male tergite 9 separated from sternite 9; posterior margin of tergite 9 varied in shape, usually with small black spines; outer gonostylus usually flattened and fleshy, and more or less acuminate; female cercus longer than hypovalva, blunt at tip, and hypovalva tapered or parallel-sided (Oosterbroek 1978; Tangelder 1983).

Xizang Autonomous Region (hereafter referred to as Xizang) is located in southwestern China and on the Qinghai-Tibet Plateau. This area is in the Palaearctic Region and on the border of the Oriental Region. With the formation and uplift of the Qinghai-Tibet Plateau, it has an average altitude of more than 4,500 m and a wide elevation range (Su et al. 2019). The formation of the Himalayas and the Qinghai-Tibet Plateau created a variety of climates and vicariance (Li and Fang 1999). Therefore, Xizang, is one of the most important biodiversity hotspots in the world. So far, only the following eight species of *Nephrotoma* are known to occur in this area: *N. claviformis* Yang & Yang, 1987, *N. didyma* Yang & Yang, 1987, *N. distans* Edwards, 1928, *N. inorata* Alexander, 1951, *N. kaulbacki* Alexander, 1951, *N. libra* Alexander, 1951, *N. nigrohalterata* Edwards, 1928, and *N. xizangensis* Yang & Yang, 1987. In the present paper, three species, including two new species, are added to the fauna of Xizang, and four species are redescribed. A key to the species of *Nephrotoma* from Xizang is presented.

## Materials and methods

The specimens were studied and illustrated with a ZEISS Stemi 2000-c stereo microscope. Details of coloration were checked in specimens immersed in 75% ethyl alcohol (C2H5OH), except the dried specimens of *N. claviformis* Yang & Yang, 1987. Genitalic preparations of males were made using lactic acid solution (C3H6O3 > 85%) heated in a water-bath to 95 °C for 4–6 minutes. After examination, it was transferred to fresh glycerin (C3H8O3) and stored in a microvial pinned below the specimen. Type specimens are deposited in the Entomological Museum of China Agricultural University (CAU), Beijing.

The morphological terminology mostly follows Alexander and Byers (1981), McAlpine (1981), Tangelder (1985), and de Jong (2017). The terminology applied to the wing veins follows the interpretation of de Jong (2017). Terminology of male hypopygium follows Alexander and Byers (1981) and Tangelder (1985).

## Taxonomy

### Key to species (males) of *Nephrotoma* from Xizang (Tibet), China

**Table:** 

1	Vertex with triangular spots near inner margin of eye; prescutum with narrow black margin (Edwards 1928: 700)	***N. distans* Edwards, 1928**
–	Vertex and prescutum not as above	**2**
2	Mediotergite with a pair of black spots; tergite 9 with black caudal margin, lateral angle produced into a decurved spine (Yang and Yang 1987: 128)	***N. libra* Alexander, 1951**
–	Mediotergite and tergite 9 not as above	**3**
3	Scutellum mainly black or dark brown	**4**
–	Scutellum mainly yellow	**5**
4	Occipital marking square, extended anteriorly to inner margin of eye (Figs 1, 2); thoracic pleuron without obvious massive black spots; tergite 9 with a deep median notch (Figs 7, 8)	***N. beibengensis* sp. nov.**
–	Occipital marking not as above; thoracic pleuron with obvious massive black spots; tergite 9 without deep median notch	**6**
5	Mediotergite and scutellum with brownish longitudinal spot at middle	**7**
–	Mediotergite and scutellum not as above	**8**
6	Lateral prescutal stripe straight; tergite 9 with a pair of rod-like projections on posterior margin (Figs 10–12)	***N. claviformis* Yang & Yang, 1987**
–	Anterior end of lateral prescutal stripe bent outward (Figs 43, 44); posterior extension of tergite 9 depressed with two pairs of short projections (Fig. 46)	***N. nigrohalterata* Edwards, 1928**
7	Posterior margin of mediotergite slightly dark; tergite 9 with a pair of large aduncous protuberances on posterior margin (Yang and Yang 1987: 128)	***N. inorata* Alexander, 1951**
–	Posterior margin of mediotergite not as above; tergite 9 with a pair of short sharp protuberances on posterior margin (Figs 27, 32)	***N. evittata* Alexander, 1935**
8	Occipital marking subtriangular; scutellum with pale brown middle stripe and brown posterior margin (Fig. 53)	***N. xizangensis* Yang & Yang, 1987**
–	Occipital marking and scutellum not as above	**9**
9	Occipital marking dark brown, bell-shaped, extended to top of vertex; tergite 9 strongly protruded at posterior margin (Figs 19, 22)	***N. didyma* Yang & Yang, 1987**
–	Occipital marking and tergite 9 not as above	**10**
10	Posterior margin of tergite 9 produced at middle, with a pair of pointed lateral projections; sternite 8 depressed at posterior margin, medially with a sickle fleshy appendage bearing long apical hairs (Figs 37, 39, 41)	***N. hanae* sp. nov.**
–	Posterior margin of tergite 9depressed at middle, without pointed lateral projections; sternite 8 with a very deep and narrow notch at posterior margin (Alexander 1953: 332)	***N. kaulbacki* Alexander, 1951**

#### Nephrotoma beibengensis

Taxon classificationAnimaliaDipteraTipulidae

sp. nov .

3A7D97C8-C3CD-5EE3-A28E-A6BDF49C2D6D

http://zoobank.org/01272B09-7641-4EAD-BA49-A378DA40D942

Figs 1–3 , 4–9 , 60

##### Diagnosis.

**Figures 1–3.**
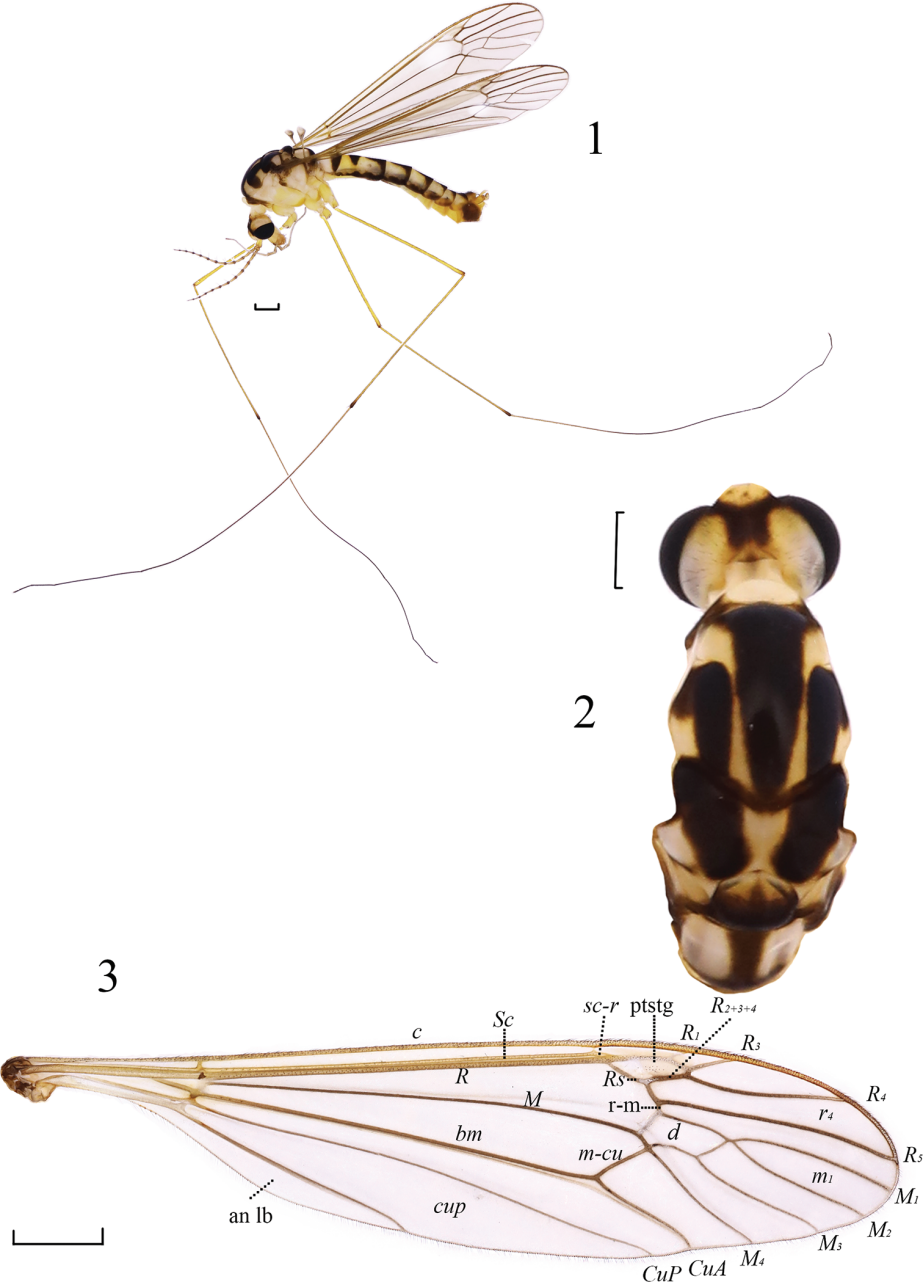
*Nephrotoma beibengensis* sp. nov. **1** Male habitus, lateral view **2** head and thorax, dorsal view **3** wing. Abbreviations: ptstg = pterostigma. Scale bars: 1.0 mm.

Frontal tubercle raised into discoid protuberance. Occipital marking dark brown, square, anteriorly extending to eye. Mediotergite with an I-shaped, dark-brown, longitudinal stripe. Legs approximately three times as long as body (Figs 1, 2). Posterior extension of tergite 9 slightly depressed with two pairs of short, obtuse projections bearing tiny, black spines, medial pair slightly longer than lateral pair (Figs 7, 8).

##### Material examined.

***Holotype*** male (CAU), China: Xizang, Motuo (Medog, Metok), Beibeng, 2017.VI.11, 859 m, Qicheng Yang (light trap). ***Paratype*** 1 male (CAU), China: Xizang, Motuo, Beibeng, Gelincun, 2018.VI.27, 1400 m, Qicheng Yang (light trap).

##### Description.

Male (*n* = 2): body length 10.4–10.5 mm, wing length 10.0 mm, antenna length 2.5 mm.

***Head*** (Figs 1, 2). Mainly yellow. Frontal tubercle raised into discoid. Occipital marking dark brown, square, extended anteriorly to inner margin of eye. Rostrum yellow and nasus brown. Hairs on head black. Antenna brownish yellow, base of each segment darker; first flagellomere 1.3 times longer than second one. Proboscis brownish yellow, with black hairs. Palpus brown, with black hairs.

***Thorax*** (Figs 1, 2). Mainly yellow. Pronotum yellow, lateral side dark brown, with yellow hairs. Prescutum with three black, longitudinal stripes; anterior end of lateral prescutal stripe bent outward, outer part brown and dull; prescutum with posterolateral dark-brown margin. Scutum with two black spots. Scutellum dark brown, with two triangular, yellow spots anteriorly. Mediotergite with an I-shaped, dark-brown marking. Anepisternum and katepisternum yellow. Anepimeron yellow with brown spot at antero-dorsal corner. Parascutellum pale brown. Anatergite brown; katatergite yellow. Meron yellow. Hairs on thorax dark yellow. Legs long, approximately three times as long as body, yellow except apices of tibiae and tarsi dark brown; hairs brown. Wing subhyaline, tinged with light brown; pterostigma pale with macrotrichiae; cell m_1_ shortly petiolate, virtually invisible; apices of R_5_ not curve up (Fig. 3). Halter with stem brownish yellow; knob pale yellow.

***Abdomen*** (Fig. 1). Mainly yellow. Tergites each with dark-brown, median spot and lateral, longitudinal stripe; median spot nearly square on tergite 1, fan-shaped on tergite 2, and those on tergite 3–7 triangular. Sternite 8 dark brown. Hairs on abdomen blackish.

**Figures 4–9.**
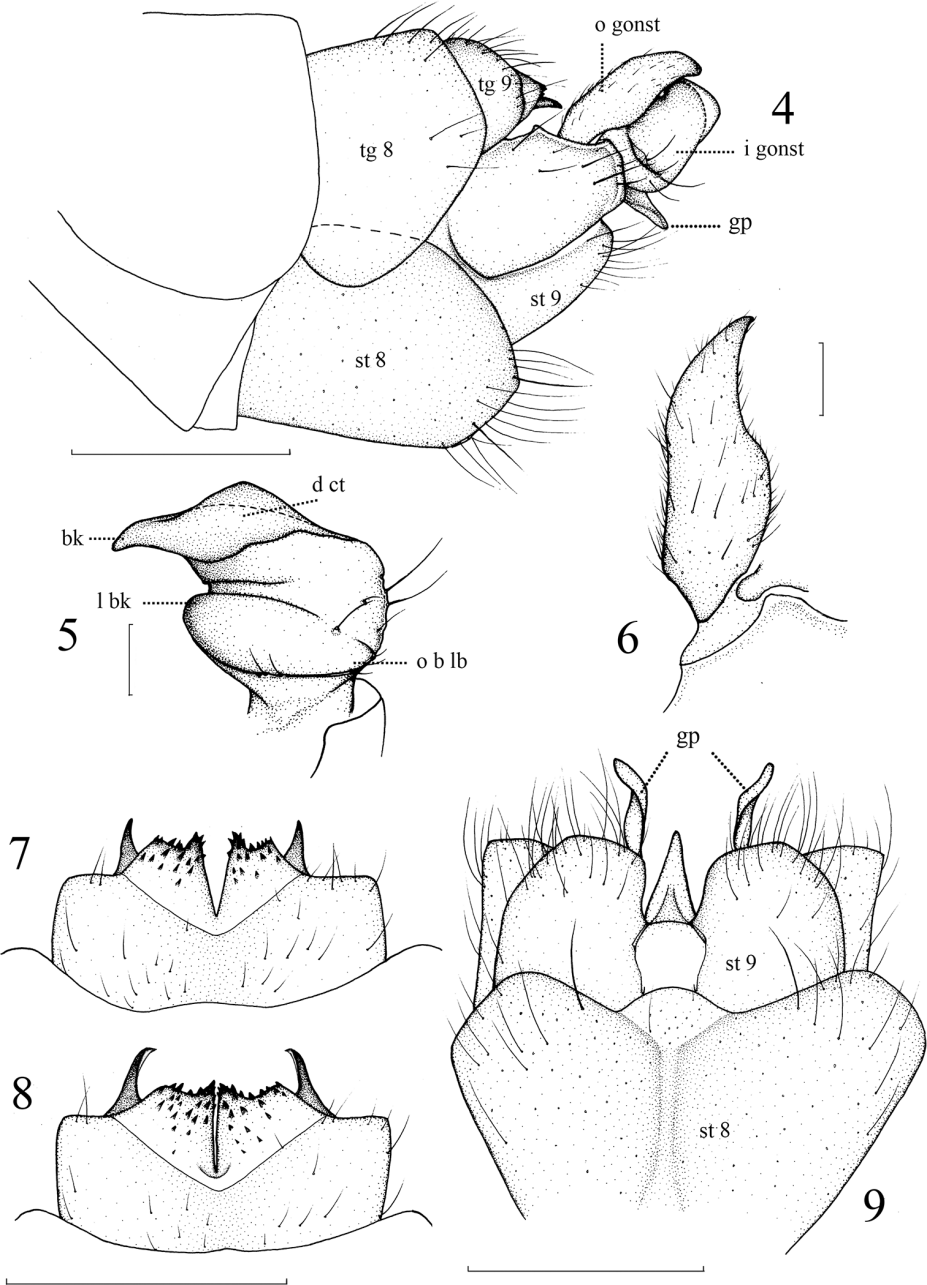
*Nephrotoma beibengensis* sp. nov. **4** Hypopygium, lateral view **5** inner gonostylus, lateral external view **6** outer gonostylus, lateral external view, before softened **7** tergite 9, dorsal view, softened **8** tergite 9, dorsal view. before softened **9** hypopygium, ventral view. Abbreviations: bk = beak, d ct = dorsal crest, gp = gonapophyses, l bk = lower beak, i gonst = inner gonostylus, o b lb = outer basal lobe, o gonst = outer gonostylus, pct= posterior crest, tg = tergite, st = sternite. Scale bars: 0.5 mm (**4, 7–9**); 0.1 mm (**5, 6**).

***Hypopygium*** (Figs 4–9) mainly brown, posterior margin yellow. Posterior margin of tergite 9 medially produced with a deep notch, apices of produced part slightly depressed, with a pair of pointed lateral projections (Figs 7, 8). Mid-posterior margin of sternite 8 depressed, with round process (Fig. 9). Gonapophyses of adminiculum twisted (Fig. 9). Outer gonostylus fleshy, small, with curly tip (Fig. 6). Inner gonostylus with large concavity at base; beak slightly obtuse, slightly produced at top (Fig. 5).

##### Distribution.

China (Xizang).

##### Remarks.

This new species is similar to *N. globate* Alexander, 1951 from India, but the latter differs in the following characters: tergite 9 with lateral projections tipped with two or three spicules; outer gonostylus long and slender; inner gonostylus long and narrow, lower beak like a slender blackened rod; sternite 8 with two tufts of hair.

##### Etymology.

This species is named after the type locality, Beibeng, Xizang, China.

#### Nephrotoma claviformis

Taxon classificationAnimaliaDipteraTipulidae

Yang & Yang, 1987

9264F8BB-7FBC-5489-AABB-B0A40D06CD36

Figs 10–11 , 12–16

Nephrotoma claviformis Yang & Yang, 1987: 129. Type locality: China: Xizang, Nyingchi Co.

##### Diagnosis.

Spots on head velvety. Vertex with rectangular, dark-brown spots near inner margin of eye. Scutellum dark brown. Mediotergite with a brown longitudinal spot at middle (Fig. 11). Tergite 9 with a pair of short rod-like projections (Fig. 12).

**Figures 10, 11.**
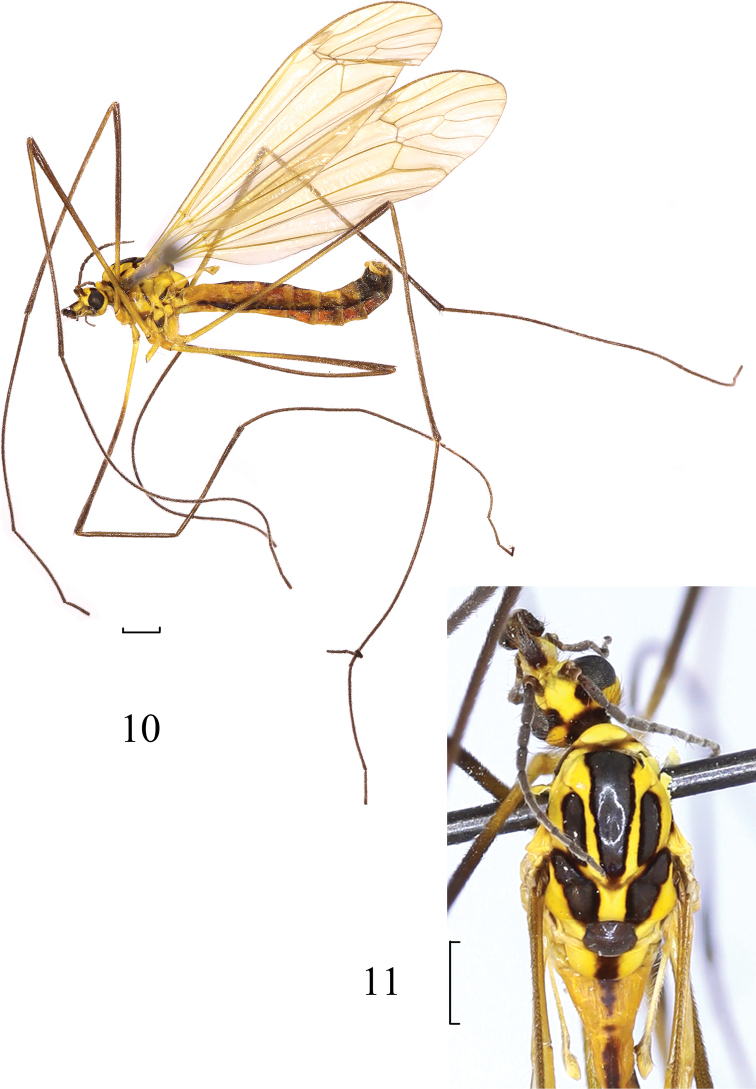
*Nephrotoma claviformis* Yang & Yang, 1987. **10** Male habitus, lateral view **11** head and thorax, dorsal view. Scale bars: 1.0 mm.

##### Material examined.

2 males (CAU), China: Xizang, Lulang, 1978.VI.10, 3400 m, Fasheng Li.

##### Description.

Males (*n* = 2): body length 11.0–11.1 mm, wing length 11.8–12.0 mm, antenna length 3.0 mm.

***Head*** (Figs 10, 11). Mainly yellow. Vertex with velvety, dark-brown, rectangular spots near inner margin of eye. Occipital marking annular, velvety, dark brown. Face with short, linear marking. Dorsal part of rostrum including nasus brownish black. Head with brown hairs. Antenna brownish black, with dark-brown hairs, except scape and pedicel dark brownish yellow; first flagellomere 1.6 times as long as second one. Proboscis brown, with dark-brown hairs. Palpus brown, with brownish-yellow hairs.

***Thorax*** (Figs 10, 11). Mainly yellow. Pronotum mainly yellow, with dark-brown spots on lateral side. Prescutum with three black, longitudinal stripes bearing velvety margin. Scutum with two black spots bearing velvety margin. Scutellum dark brown. Mediotergite yellow, with a brown, velvety, longitudinal stripe at middle. Anepisternum and katepisternum each with a brown lower spot and black posterior margin. Anepimeron and katepimeron each with a small, pale-brown spot. Meron with black lower part. Parascutellum yellow, anatergite yellow, katatergite with black posterior margin. Legs dark yellow except tips of tibiae, femora, and tarsi dark brown. Hairs on legs brownish, except those on coxae yellow. Wing subhyaline, tinged with light brown; pterostigma brownish; vein of cell m_1_ sessile (Fig. 10). Halter with yellow stem; knob dark yellow.

***Abdomen*** (Fig. 10). Mainly yellow. Abdominal tergites with brown mid-longitudinal stripe and brownish-black lateral stripe. Hairs on abdomen yellow.

**Figures 12–16.**
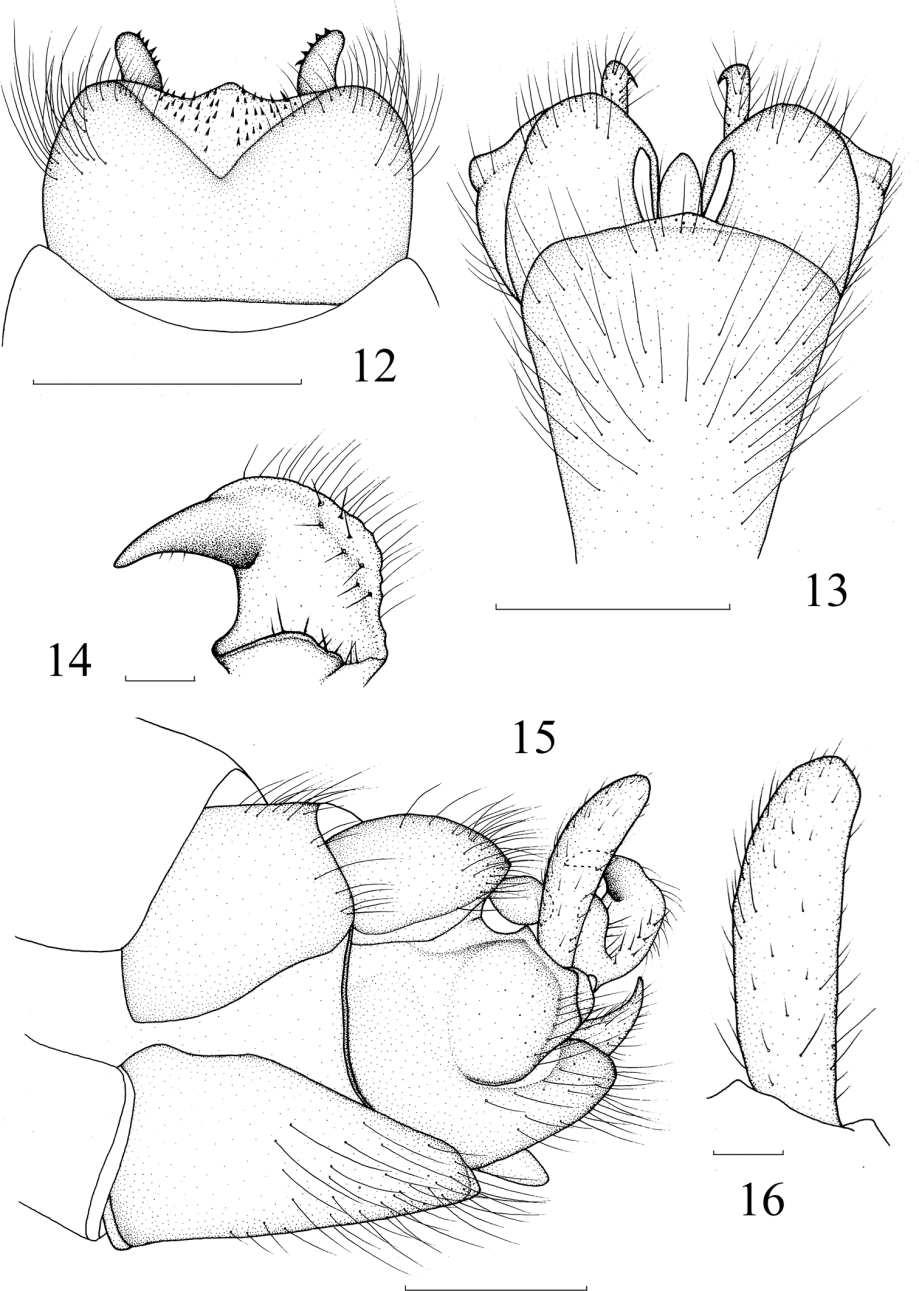
*Nephrotoma claviformis* Yang & Yang, 1987. **12** Tergite 9, dorsal view **13** hypopygium, ventral view **14** inner gonostylus, lateral external view **15** hypopygium, lateral view **16** outer gonostylus, lateral external view. Scale bars: 0.5 mm (**12, 13, 15**); 0.1 mm (**14, 16**).

***Hypopygium*** (Figs 12–16) brownish black. Tergite 9 medially slightly produced at posterior margin, tergite 9 with a pair of rod-like projections on posterior margin (Fig. 12). Outer gonostylus short rod-like (Fig. 16). Inner gonostylus anteriorly with a sharp beak at tip, laterally with a lobe at base (Fig. 14).

##### Distribution.

China (Xizang).

##### Remarks.

This species is similar to *N. distans* Edwards, 1928 from Xizang, the inner gonostylus is similar, but the latter differs in the following characters: occipital marking triangular, scutellum and postnotum pale; tergite 9 without rod-like projections on posterior margin.

#### Nephrotoma didyma

Taxon classificationAnimaliaDipteraTipulidae

Yang & Yang, 1987

BB30CF7F-6719-52AC-9F14-18765B360747

Figs 17–19 , 20–25

Nephrotoma didyma Yang & Yang, 1987: 131. Type localty: China: Xizang, Mainling Co.

##### Diagnosis.

**Figures 17–19.**
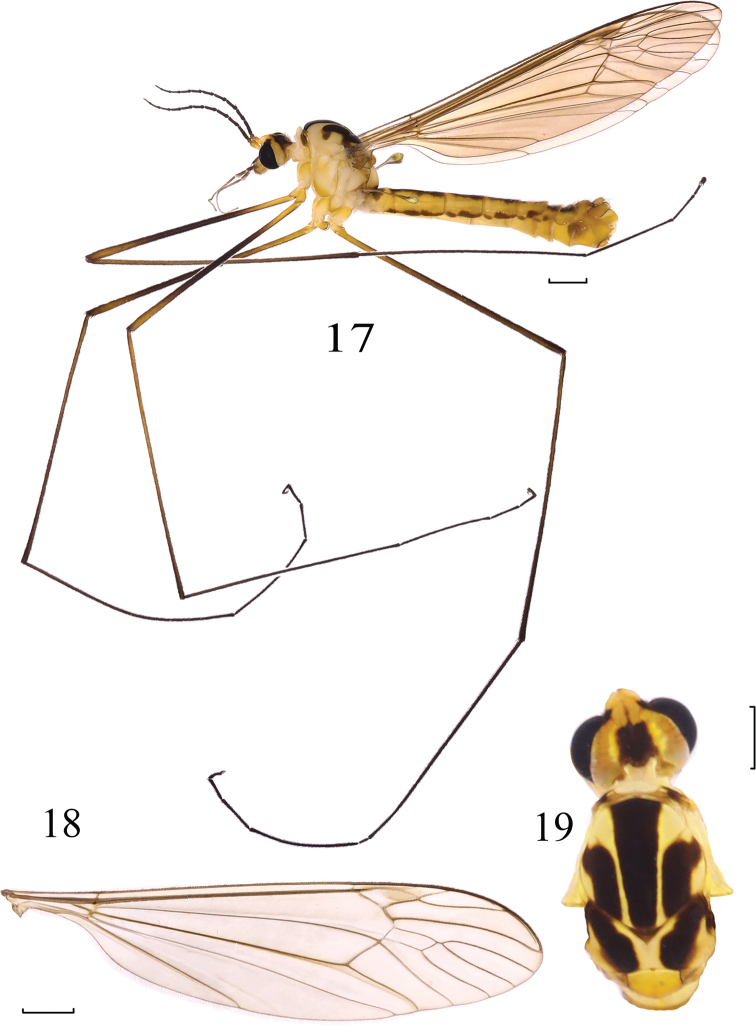
*Nephrotoma didyma* Yang & Yang, 1987. **17** Male habitus, lateral view **18** wing **19** head and thorax, dorsal view. Scale bars: 1.0 mm.

**Figures 20–25.**
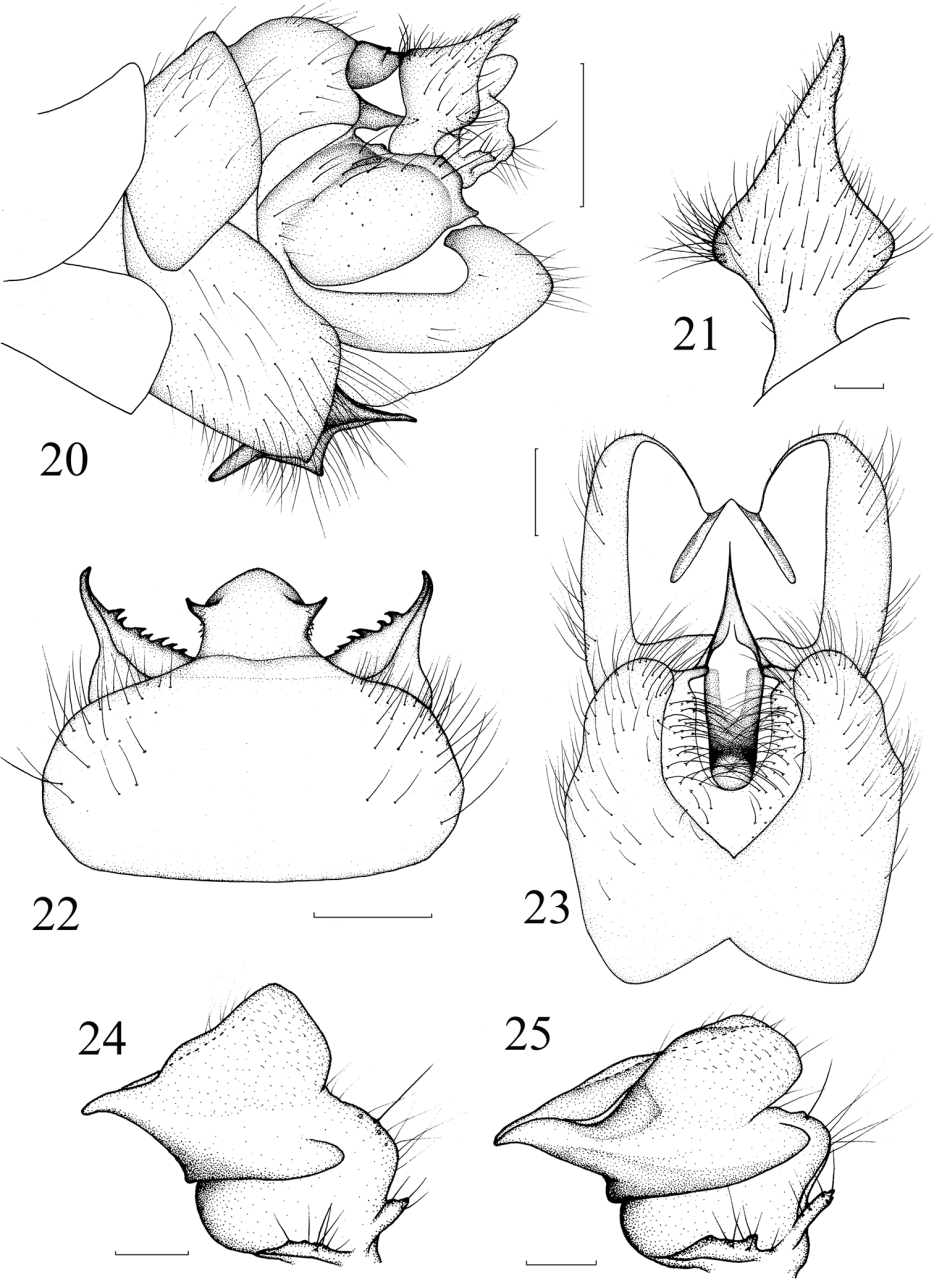
*Nephrotoma didyma* Yang & Yang, 1987. **20** Hypopygium, lateral view **21** outer gonostylus, lateral external view **22** tergite 9, dorsal view, softened **23** hypopygium, ventral view **24** inner gonostylus, lateral external view, softened **25** inner gonostylus, lateral external view, before softened. Scale bars: 0.5 mm (**20, 22, 23**); 0.1 mm (**21, 24, 25**).

Pronotum yellow with a brownish spot on lateral side. Mediotergite yellow with dark-brown posterior margin (Figs 17, 19). Posterior margin of tergite 9 with a jaw-shaped protuberance; spade-shaped protuberance with two short horns at posterior margin (Fig. 22). Posterior margin of sternite 8 with meniscus-shaped protrusion (Figs 20, 23).

##### Material examined.

4 males (CAU), China: Xizang, Yigong, 2017.VI.7, 2236 m, Qicheng Yang (light trap). 3 males (CAU), China: Xizang, Yigong, 2017.V 6, 2274 m, Qicheng Yang (light trap). 6 males (CAU), China: Xizang, 106K, 2017.V.17, 2289 m, Qicheng Yang (light trap).

##### Description.

Male (*n* = 13): body length 9.5–12.0 mm, wing length 10.0–12.0 mm, antenna length 3.0–4.0 mm.

***Head*** (Figs 17, 19). Mainly yellow. Vertex with brown spot near inner margin of eye. Occipital marking dark brown, bell-shaped, extended to top of vertex. Frontal tubercle relatively high. Dorsal part of rostrum including nasus brownish black. Hairs on head dark brown. Antenna dark brown except scape yellow and pedicel dark yellow; first flagellomere 1.4 times longer than second one. Proboscis yellow, with brown hairs. Palpus brownish grey, with brown hairs.

***Thorax*** (Figs 17, 19). Mainly yellow. Pronotum yellow with a brownish spot on lateral side. Prescutum with three black longitudinal stripes; anterior end of lateral prescutal stripe bent outward, outer part brown. Scutum with two large, dark-brown spots. Scutellum dark yellow. Mediotergite yellow with dark-brown posterior margin. Anepisternum and katepisternum pale yellow with yellow lower part. Anepimeron pale yellow. Parascutellum pale brown; anatergite and katatergite pale yellow. Legs yellow except middle portions of femora, tips of tibiae, and tarsi dark brown; hairs dark brown, except those on coxae and trochanters yellow. Wing subhyaline, tinged with light brown; pterostigma pale brown; cell m_1_ nearly sessile (Fig. 18). Halter with stem brownish grey; knob yellowish brown.

***Abdomen*** (Fig. 17). Mainly yellow. Abdominal tergites with three brown longitudinal stripes. Hypopygium mainly dark yellow. Abdomen with yellow hairs.

***Hypopygium*** (Figs 20–25) brownish yellow. Tergite 9 ellipse with spade-shaped protuberance bearing two short horns at posterior margin; posterolateral margin of tergite 9 with a large jaw-shaped protuberance (Fig. 22). Posterior margin of sternite 8 with obvious appendages, meniscus-shaped in lateral view, covered with dense yellow hairs (Fig. 20). Outer gonostylus fleshy, spade-shaped, wide at middle, narrowed toward tip (Fig. 21). Inner gonostylus with large concavity, basally with a posterior projection (Figs 24, 25).

##### Distribution.

China (Xizang).

##### Remarks.

Hypopygium of this species is very specific; no similar species.

#### Nephrotoma distans

Taxon classificationAnimaliaDipteraTipulidae

Edwards, 1928

8EBE13BB-5980-5094-BB63-33A2CA289B4C

Nephrotoma distans Edwards, 1928: 700. Type locality: China: Tibet, Rongshar Valley.

##### Diagnosis.

Vertex with triangular black spot near inner margin of eye. Prescutum with narrow black margin. Scutellum dark. Wing completely brownish. Gonapophyses of adminiculum hooked (Edwards 1928).

##### Distribution.

China (Sichuan and Yunnan).

#### Nephrotoma evittata

Taxon classificationAnimaliaDipteraTipulidae

Alexander, 1935

8B14DBEB-D4E5-54D4-9AAF-B1F75321A424

Figs 26–28 , 29–33

Nephrotoma evittata Alexander, 1935: 200. Type locality: China: Szechwan, Shin-Kai-Si, Mount Omei.

##### Diagnosis.

**Figures 26–28.**
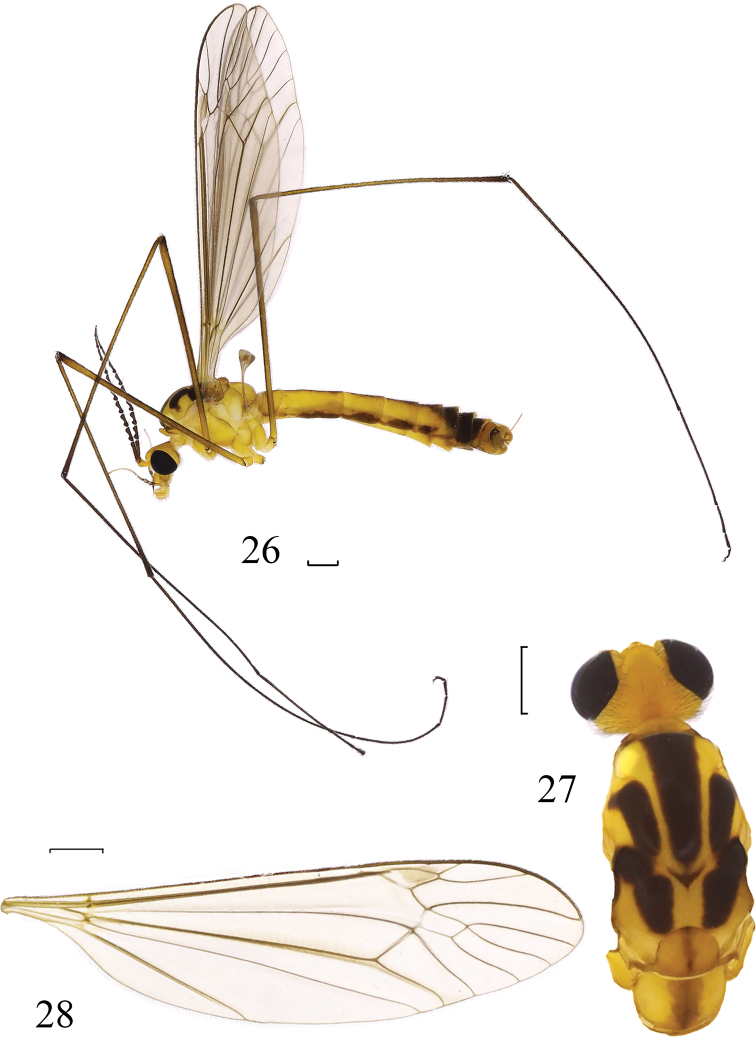
*Nephrotoma evittata* Alexander, 1935. **26** Male habitus, lateral view **27** head and thorax, dorsal view **28** wing. Scale bars: 1.0 mm.

Antenna mainly dark brown except scape yellow, pedicel and first flagellomere brown; both ends of flagellomere with obvious expansion (Fig. 26). Scutellum dark yellow, with brown middle stripe. Mediotergite yellow with brown to dark-yellow middle stripe (Fig. 27). Tergite 9 semicircular, depressed at middle, with a pair of sharp short protuberances at posterior margin (Fig. 32). Inner gonostylus with flat lower beak bearing bristles (Fig. 31).

##### Material examined.

2 males (CAU), China: Xizang, Yigong, 2017.VI.7, 2236 m, Qicheng Yang (light trap). 2 males (CAU), China: Xizang, Yigong, 2017.VIII.5, 2183 m, Qicheng Yang (light trap). 1 male (CAU), China: Xizang, 80K, 2017.VI.13, 2023 m, Qicheng Yang (light trap). 1 male (CAU), China: Xizang, 106K, 2017.V.17, 2289 m, Qicheng Yang (light trap).

##### Description.

Male (*n* = 6): body length 11.0–12.5 mm, wing length 11.0–12.0 mm, antenna length 2.5–3.5 mm.

***Head*** (Figs 26, 27). Mainly yellow. Occipital marking brown, faint. Frontal tubercle relatively high. Dorsal part of rostrum, including nasus, dark brown. Head with black hairs. Antenna dark brown except scape yellow, pedicel and first flagellomere brown; first flagellomere 1.2 times as long as second one; both ends of flagellomere obvious expansion. Proboscis mainly yellow, with black hairs. First palpal segment yellow, second one brown, with black hairs.

***Thorax*** (Figs 26, 27). Mainly yellow. Pronotum yellow, pale brown on lateral side. Prescutum with three black longitudinal stripes; anterior end of lateral prescutal stripe bent outward, outer part brown. Scutum with four black spots. Scutellum dark yellow, with brown middle stripe. Mediotergite yellow, with brown to dark-yellow middle stripe. Anepisternum and katepisternum yellow; katepisternum with yellow lower part. Anepimeron pale yellow, lower part dark yellow. Anatergite and katatergite yellow. Parascutellum yellow. Legs yellow except tips of tibiae brown and tarsi dark brown; hairs dark brown except those on coxae dark yellow. Wing subhyaline, tinged with light brown; pterostigma slightly deepened; cell m_1_ shortly petiolate (Fig. 28). Halter with stem pale brown; knob pale yellow.

***Abdomen*** (Fig. 26). Mainly yellow. Abdominal tergites with three brown longitudinal stripes. Abdominal segments 6–8 entirely dark brown to black; hypopygium mainly yellow; tergite 9 brown or yellow. Hairs on abdomen blackish or yellow.

**Figures 29–33.**
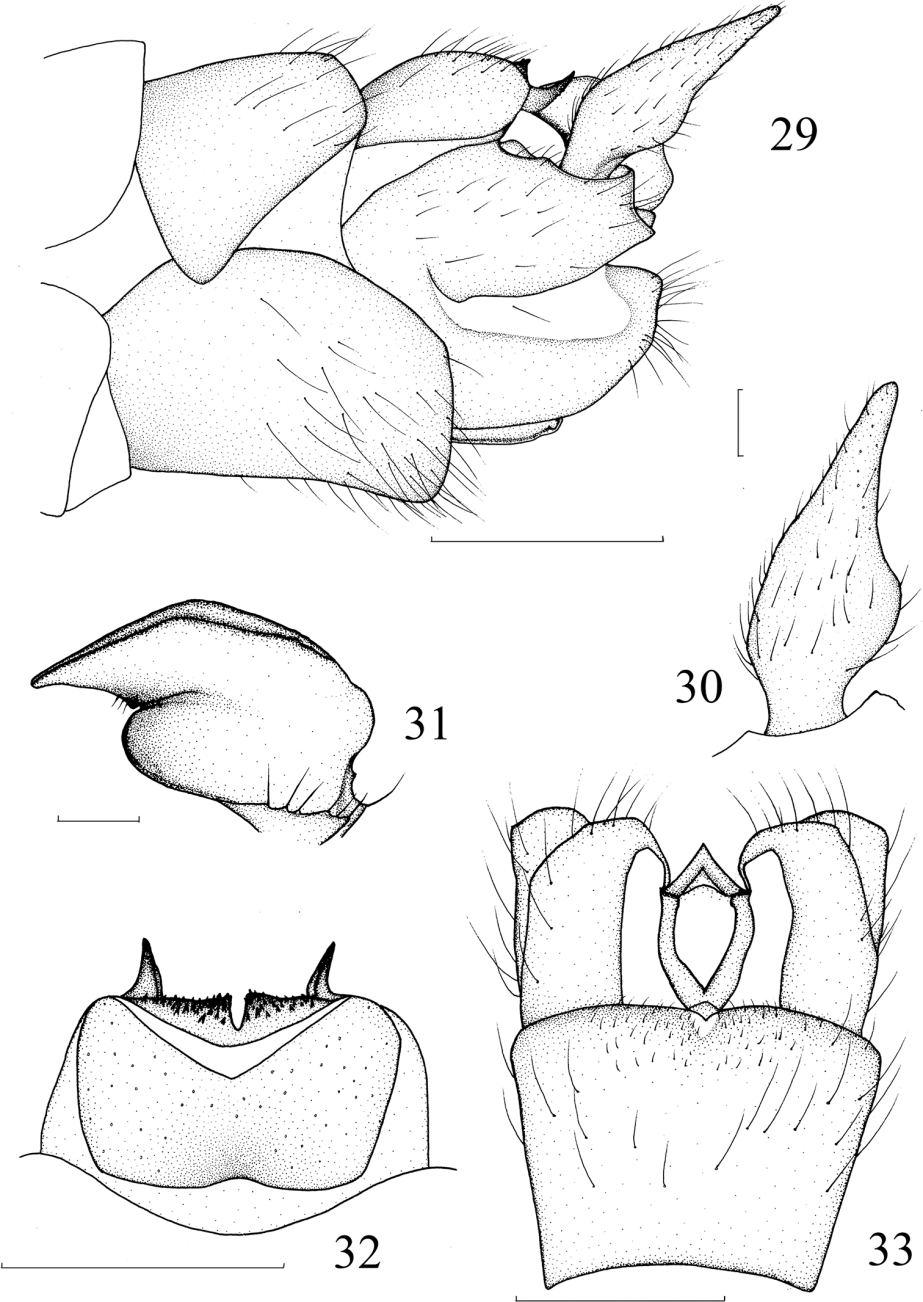
*Nephrotoma evittata* Alexander, 1935. **29** Hypopygium, lateral view **30** outer gonostylus, lateral external view **31** inner gonostylus, lateral external view **32** tergite 9, dorsal view **33** hypopygium, ventral view. Scale bars: 0.5 mm (**29, 32, 33**); 0.1 mm (**30, 31**).

***Hypopygium*** (Figs 29–33) brownish yellow. Tergite 9 semicircular, depressed at middle, with a pair of sharp short protuberances on posterior margin (Fig. 32). Posterior margin of sternite 8 with dense bristles at middle (Fig. 33). Outer gonostylus fleshy, protruded at middle, narrowed toward tip (Fig. 30). Inner gonostylus flat, with large concavity at base; lower beak with bristles (Fig. 31).

##### Distribution.

China (Sichuan, Yunnan and Xizang).

##### Remarks.

First record for Xizang. This species is similar to *N. impigra impigra* Alexander, 1935 from China (Hubei, Sichuan, Zhejiang, Fujian, Guizhou, Jiangxi), but the latter differs in the following characters: occipital marking distinct; scutellum dark brown. The hypopygium of this species is consistent with holotype, and we found that its occipital marking is variable. We think the difference between the veins is caused by the origin, which is also variable.

#### Nephrotoma hanae sp. nov.

Taxon classificationAnimaliaDipteraTipulidae

7430984B-8E54-5B1D-997D-745663AE10D0

http://zoobank.org/25F93152-725A-41E5-913D-A266A4FFF631

Figs 34–36 , 37–42

##### Diagnosis.

**Figures 34–36.**
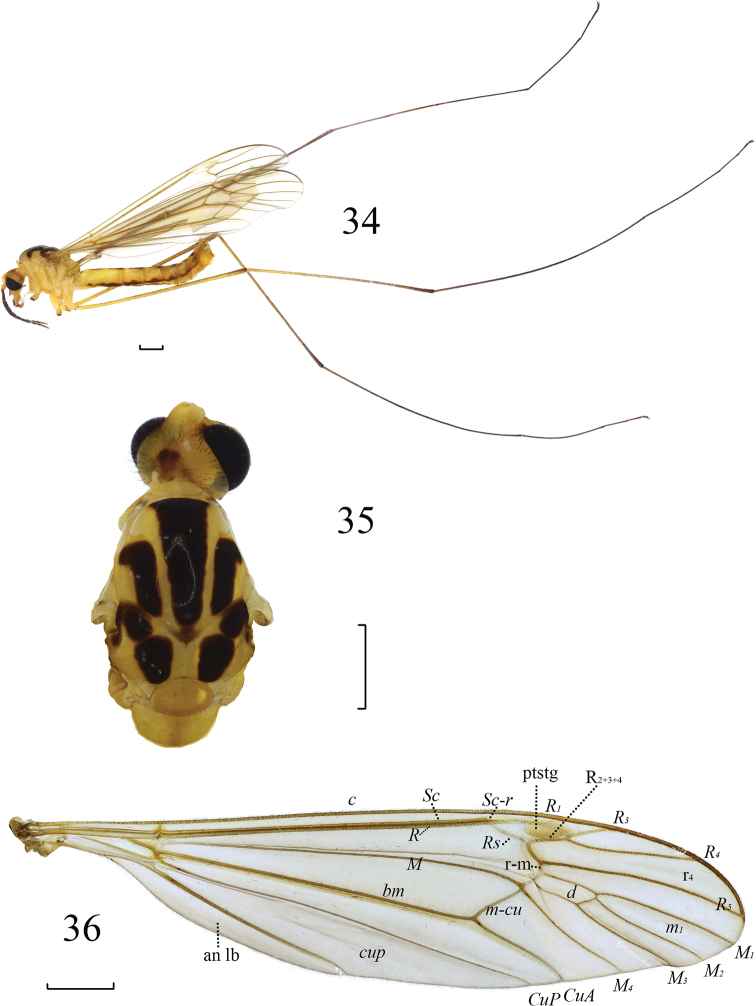
*Nephrotoma hanae* sp. nov. **34** Male habitus, lateral view **35** head and thorax, dorsal view **36** wing. Abbreviations: ptstg = pterostigma. Scale bars: 1.0 mm.

**Figures 37–42.**
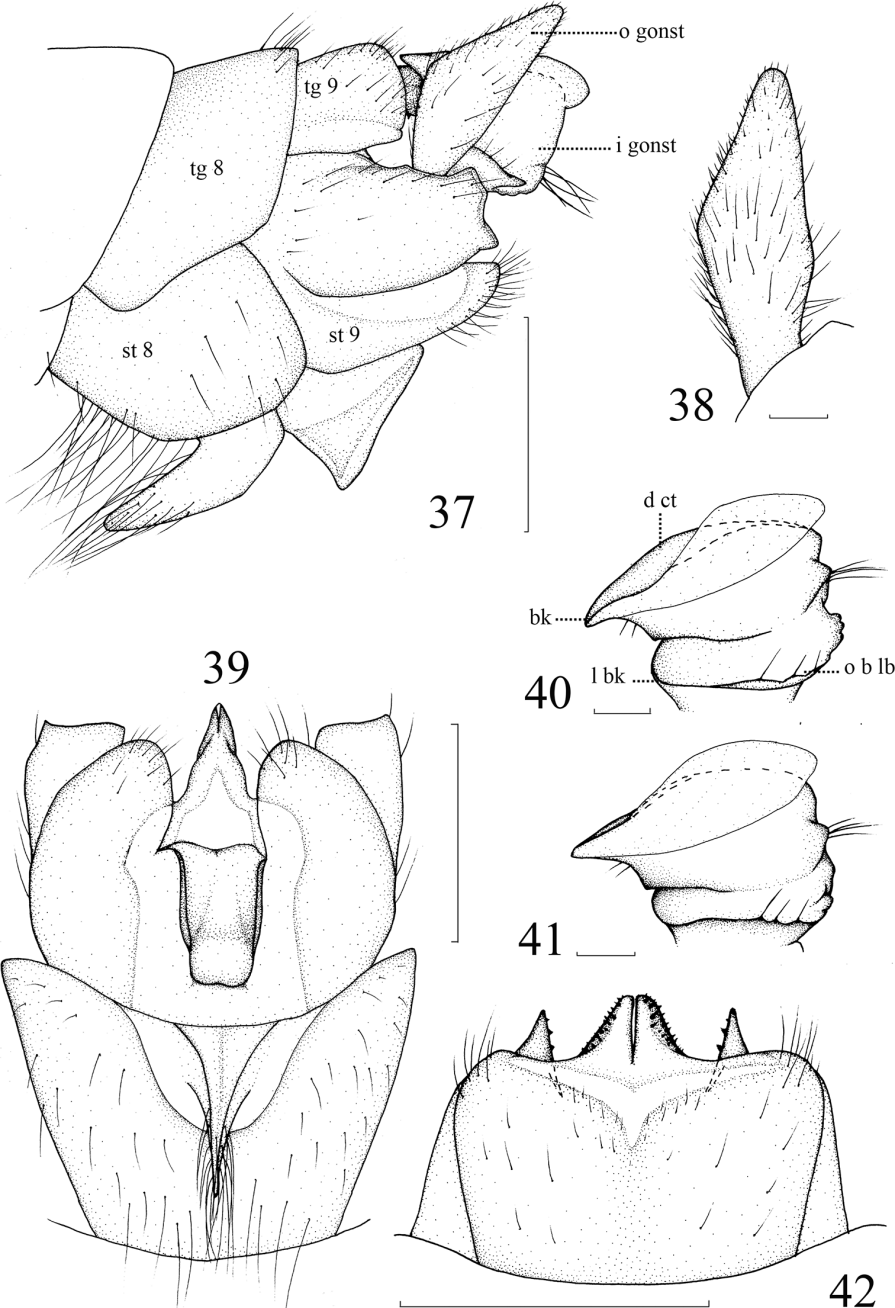
*Nephrotoma hanae* sp. nov. **37** Hypopygium, lateral view **38** outer gonostylus, lateral external view **39** hypopygium, ventral view **40, 41** inner gonostylus, lateral external view **42** tergite 9, dorsal view. Abbreviations: bk = beak, d ct = dorsal crest, l bk = lower beak, i gonst = inner gonostylus, o b lb = outer basal lobe, o gonst = outer gonostylus, tg = tergite, st = sternite. Scale bars: 0.5 mm (**37, 39, 42**); 0.1 mm (**38, 40, 41**).

Occipital marking variable, small, brown. Antennal scape yellow, pedicel brown, flagellum brownish black (Figs 34, 35). Tergite 9 medially produced with a pair of projections and a subequal notch at posterior margin; laterally with a pair of pointed projections with spines (Fig. 42). Sternite 8 depressed at posterior margin, medially with a sickle-shaped, fleshy appendage bearing long apical hairs (Figs 37, 39). Outer gonostylus slightly expanded anteriorly (Fig. 38). Inner gonostylus with a weak protuberance at posterior margin (Figs 40, 41).

##### Material examined.

***Holotype*** male (CAU), China: Xizang, Bomi (Pome), 2016.VII.12–26, 2700 m, Shaolin Han (light trap). ***Paratype***: male (CAU), China: Xizang, Bomi, Bagai, 2018.VII.1–2, 2823 m, Qicheng Yang (light trap). 1 male, 1 female (CAU), China: Xizang, Yadong, 2018.VII.12, 4000 m, Yajun Zhu. 1 male, 2 female (CAU), China: Xizang, Bomi, Bagai, 2018.VII.1–2, 2823 m, Qicheng Yang (light trap).

##### Description.

Male (*n* = 4): body length 10.3–10.6 mm, wing length 11.0–11.6 mm, antenna length 2.8–3.0 mm.

***Head*** (Figs 34, 35). Mostly yellow. Occipital marking variable, small, brown. Frontal tubercle relatively high. Rostrum and nasus brown. Hairs on head dark brown. Antennal scape yellow, pedicel brown, flagellum brownish black; first flagellomere 1.3 times longer than second segment. Proboscis brown, with dark-brown hairs. Palpus brownish yellow, with dark-brown hairs.

***Thorax*** (Figs 34, 35). Mostly yellow. Pronotum yellow, with a small, pale-brown spot on lateral side. Prescutum with three black longitudinal stripes; anterior end of lateral prescutal stripe bent outward, outer part brown. Scutum with four brownish-black spots, anterior margin of lateral spot brown. Scutellum and mediotergite yellow. Pleuron pale yellow, but lower part of katepisternum and katatergite yellow. Parascutellum yellow. Legs yellow, except tips of femora dark yellow, tips of tibiae and tarsi dark brown; hairs brown, except those on coxae and trochanters yellow. Wing subhyaline, tinged with light brown; pterostigma brown; cell m_1_ sessile, cell m_1_ and cell d narrow; apices of R_5_ not curving up (Fig. 36). Halter pale yellowish brown.

***Abdomen*** (Fig. 34). Mainly yellow. Abdominal tergites each with three brown spots, lateral spots rather narrow and fused with each other; tergite 1 without spot, median spot on tergites 2 and 3 roughly rectangular, on tergites 4–8 subtriangular. Abdomen with yellow and brown hairs.

***Hypopygium*** (Figs 37–42) mainly dark yellow. Tergite 9 medially produced, with notch, lateral side with a pointed projection with spines (Fig. 42). Sternite 8 depressed at posterior margin, medially with a sickle-shaped, fleshy appendage bearing long apical hairs (Figs 37, 39). Sternite 9 with a subtriangular appendage (Figs 37, 39). Outer gonostylus fleshy, small, anteriorly slightly expanded, apically pointed (Fig. 38). Inner gonostylus with concavity at base; beak sharp, posterior margin with a small protuberance with five bristles (Figs 40, 41).

##### Distribution.

China (Xizang).

##### Remarks.

This new species is similar to *N. korpa* Alexander, 1967 from India (Sikkim), but the latter differs in the following characters: posterior vertex with three brown spots at narrowest point; projections of tergite 9 smaller, but lateral projections without spines; outer gonostylus unusually long, basally expanded.

##### Etymology.

This species is named after the collector, Shaolin Han.

#### Nephrotoma inorata

Taxon classificationAnimaliaDipteraTipulidae

Alexander, 1951

CFD70061-CA1A-5F32-967C-EBB18E7F6D4A

Nephrotoma inorata Alexander, 1951: 1096. Type locality: China: south-eastern Tibet, Eong To Valley.

##### Diagnosis.

Frontal tubercle slightly forked, clover-shaped. Scutum and scutellum with brownish longitudinal spot at middle. Posterior margin of mediotergite slightly darker. Sternite 8 with long hairs, but without process at posterior margin (Alexander 1951, 1967).

##### Distribution.

China (Xizang), India (W Bengal).

#### Nephrotoma kaulbacki

Taxon classificationAnimaliaDipteraTipulidae

Alexander, 1951

7E4198BF-B8B7-5B99-962D-DFD0FF8E51D7

Nephrotoma kaulbacki Alexander, 1951: 1094. Type locality: China: eastern Tibet, Poshö, Kyari Dzong.

##### Diagnosis.

Antennal scape and pedicel pale yellow, flagellum black. Scutellum and mediotergite without spot. Tergite 9 with a deep median notch. Sternite 8 with a very deep and narrow notch at posterior margin. Gonapophyses of adminiculum hooked (Alexander 1951, 1953).

##### Distribution.

China (Xizang).

#### Nephrotoma libra

Taxon classificationAnimaliaDipteraTipulidae

Alexander, 1951

CF4F681E-DD73-54B5-ADC4-3E0435676EF2

Nephrotoma libra Alexander, 1951: 1092. Type locality: China: Tibet, Gyantse.

##### Diagnosis.

Antennal scape reddish brown, pedicel blackish brown, flagellum black. Mediotergite with a pair of black spots. Tergite 9 with thick, black caudal margin, lateral angle produced into a decurved spine. Sternite 8 without process. Sternite 9 with a finger-like process. Gonapophyses of adminiculum hooked (Alexander 1951, 1953).

##### Distribution.

China (Xizang).

#### Nephrotoma nigrohalterata

Taxon classificationAnimaliaDipteraTipulidae

Edwards, 1928

4984D93C-7567-56F6-ACA8-E5D2DC21DAFF

Figs 43–45 , 46–51 , 62

Nephrotoma nigrohalterata Edwards, 1928: 700. Type locality: China: Szechwan-Tibet border, Yien-Long-Shien.
Nephrotoma attenuata Alexander, 1935: 135. Type locality: China: Szechwan-Tibet border, Yien-Long-Shien.

##### Diagnosis.

**Figures 43–45.**
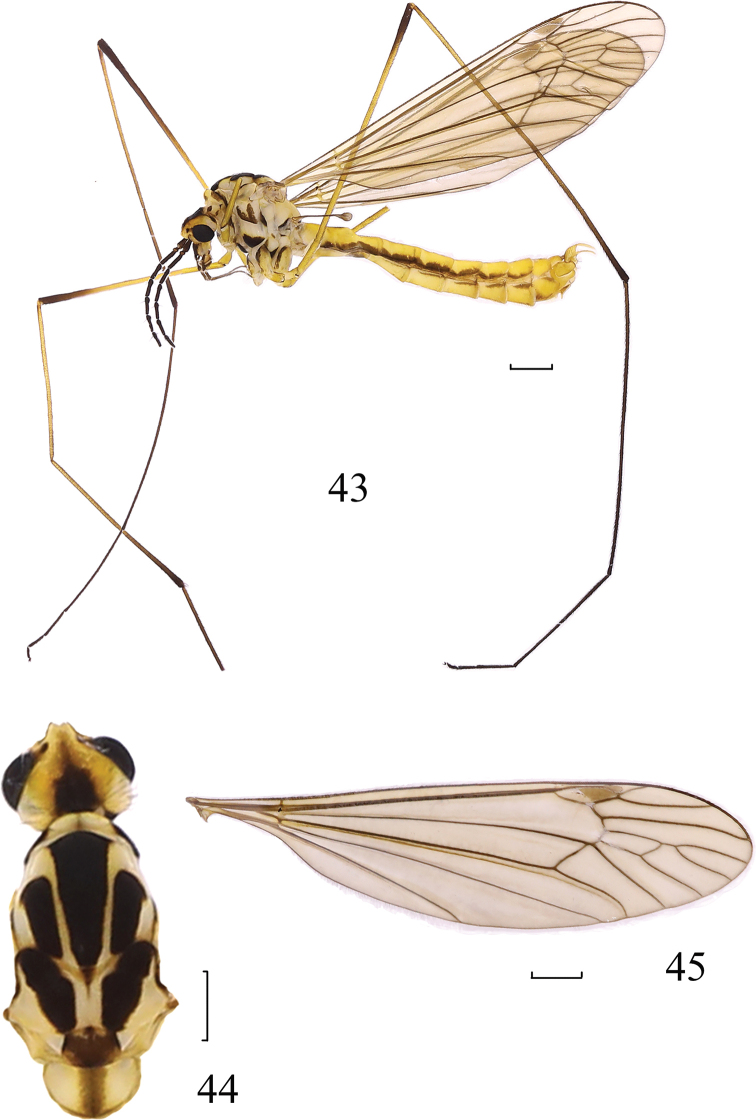
*Nephrotoma nigrohalterata* Edwards, 1928. **43** Male habitus, lateral view **44** head and thorax, dorsal view **45** wing. Scale bars: 1.0 mm.

**Figures 46–51.**
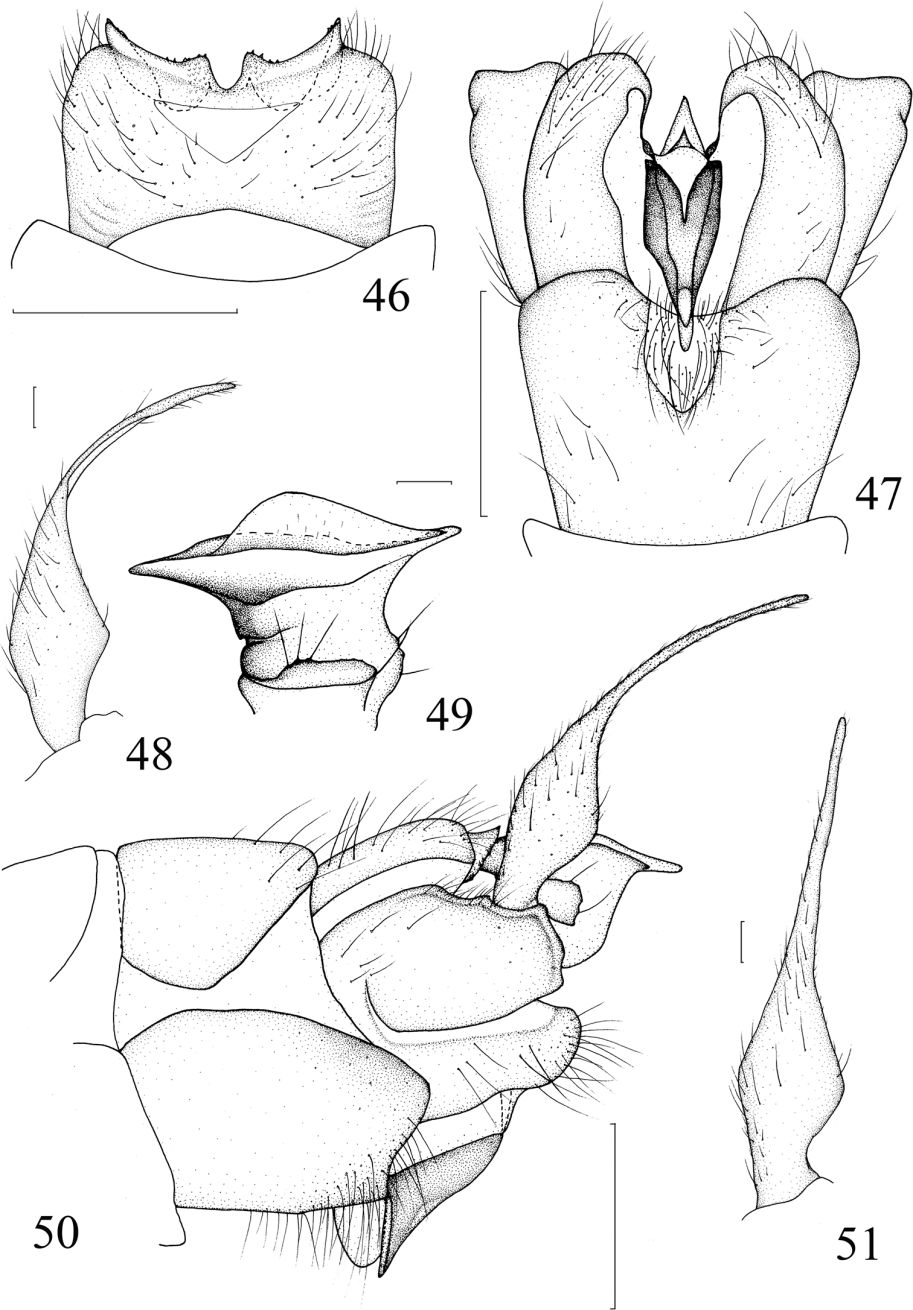
*Nephrotoma nigrohalterata* Edwards, 1928. **46** Tergite 9, dorsal view, softened **47** hypopygium, ventral view **48** outer gonostylus, lateral external view, before softened **49** inner gonostylus, lateral external view **50** hypopygium, lateral view **51** outer gonostylus, lateral external view, softened. Scale bars: 0.5 mm (**46, 47, 50**); 0.1 mm (**48, 49, 51**).

Vertex with round, dark-brown spot near inner margin of eye; dorsal part of rostrum including nasus brownish black. Antenna black (Figs 43, 44). Tergite 9 distinctly depressed (Fig. 46). Sternite 9 with a brown, horn-like projection. Outer gonostylus greatly produced and slender (Figs 47, 50).

##### Material examined.

12 male (CAU), China: Xizang, Bayi, 2017.VI.2, 2950 m, Qicheng Yang (light trap).

##### Description.

Male (*n* = 12): body length 10.5–12.5 mm, wing length 10.5–12.5 mm, antenna length 3.5–5.0 mm.

***Head*** (Figs 43, 44). Mainly yellow. Vertex with round, dark-brown spot near inner margin of eye. Occipital marking dark brown, subtriangular. Face with obvious linear marking. Posterior margin of postgena brown. Dorsal part of rostrum including nasus brownish black. Head with black hairs. Antenna black with dense villi; first flagellomere 1.2 times longer than second one. Proboscis mainly yellow, with brown hairs. Palpus greyish brown, with brown hairs.

***Thorax*** (Figs 43, 44). Mainly yellow. Pronotum mainly yellow, with large, brown spots on lateral side. Prescutum with three black longitudinal stripes, middle stripe extended to scutum, anterior end of lateral prescutal stripe slightly bent outward. Scutum with two black spots. Scutellum dark brown. Mediotergite with a brown longitudinal stripe at middle. Anepisternum and katepisternum each with large black spot, posterior margin of anepisternum and katepisternum black; spot of anepisternum tilted V-shaped. Anepimeron and katepimeron each with small pale brown spot. Meron with black lower portion. Anatergite yellow, katatergite with black lower margin. Parascutellum yellow. Legs yellow, except anterior margin of coxae brown black, tips of tibiae and femora brown; hairs brownish except those on coxae yellow. Wing subhyaline, tinged with light brown; pterostigma greyish brown; vein of cell m_1_ shortly petiolate, cell d almost as long as cell m_1_ (Fig. 45). Halter with stem brown; knob yellowish brown.

***Abdomen*** (Fig. 43). Mainly yellow. Abdominal tergites with three dark-brown longitudinal stripes. Abdomen with yellow hairs.

***Hypopygium*** (Figs 46–51) yellow. Posterior extension of tergite 9 depressed, with two pairs of short projections. Posterior margin of sternite 8 depressed, medially with long hairs, produced ventrad into a small, pale, fleshy lobe (Figs 46, 47). Sternite 9 with a brown, horn-like projection (Figs 47, 50). Outer gonostylus greatly produced and slender (Figs 48, 51). Inner gonostylus with sharp beak, posterior crest produced backward; outer basal lobe with three strong, black setae (Fig. 49).

##### Distribution.

China (Sichuan, Xizang)

##### Remarks.

This species is similar to *N. geniculata* Yang & Yang, 1987 from China (Hubei, Inner Mongolia, Ningxia, Sichuan), but the latter differs in the following characters: without occipital marking; dorsal part of rostrum including nasus yellow. Posterior margin of sternite 8 undepressed, without fleshy lobe ventrally.

#### Nephrotoma xizangensis

Taxon classificationAnimaliaDipteraTipulidae

Yang & Yang, 1987

A0B0EF9F-CD62-5240-BF20-5181A3242A34

Figs 52–54 , 55–59 , 61

Nephrotoma xizangensis Yang & Yang, 1987: 129. Type locality: China: Xizang, Nyingchi Co.

##### Diagnosis.

**Figures 52–54.**
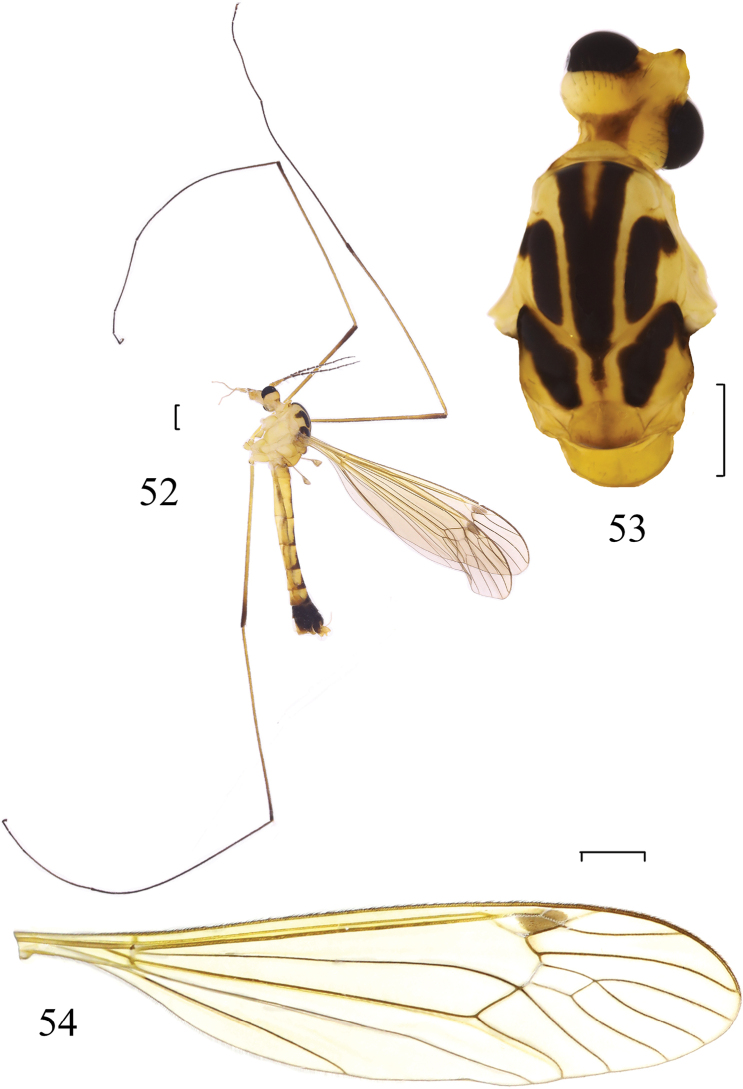
*Nephrotoma xizangensis* Yang & Yang, 1987. **52** Male habitus, lateral view **53** head and thorax, dorsal view **54** wing. Scale bars: 1.0 mm.

Antennal scape yellow, pedicel pale brown, flagellum mainly dark brown, except first flagellomere pale brown (Fig. 52). Mid-longitudinal stripe of prescutum V-shaped. Scutellum with pale brown middle stripe and brown posterior margin. Mediotergite yellow (Fig. 53). Posterior extension of tergite 9 slightly depressed, with two pairs of short obtuse projections (Fig. 58).

##### Material examined.

2 males (CAU), China: Xizang, Yigong, 2017.VI.7, 2236 m, Qicheng Yang (light trap). 1 male (CAU), China: Xizang, Bayi, 2017.VI.4, 2955 m, Qicheng Yang (light trap).

##### Description.

Male (*n* = 3): body length 11.5–12.5 mm, wing length 11.5–12.5 mm, antenna length 3.5–4.0 mm.

***Head*** (Figs 52, 53). Mainly yellow. Vertex with brown spot near inner margin of eye. Occipital marking brown, subtriangular. Dorsal part of rostrum including nasus brown. Head with black hairs. Antennal scape yellow, pedicel pale brown, flagellum mainly dark brown except first flagellomere pale brown; first flagellomere 1.5 times longer than second one. Proboscis yellow, with brown hairs. First palpal segment greyish brown, second one yellow, with brown hairs.

***Thorax*** (Figs 52, 53). Mainly yellow. Pronotum yellow. Prescutum with three black longitudinal stripes; middle stripe V-shaped, anterior end of lateral prescutal stripe bent outward, outer curved part brown. Scutum yellow, with two black subtriangular spots. Scutellum with pale brown middle stripe and brown posterior margin. Mediotergite yellow. Pleuron pale yellow. Parascutellum yellow. Legs yellow, except tips of femora and tibiae brown, tarsi dark brown; hairs dark brown. Wing subhyaline, tinged with light brown; pterostigma brown; cell m_1_ sessile (Fig. 54). Halter brownish grey.

***Abdomen*** (Fig. 52). Mainly yellow. Abdominal tergites with three longitudinal stripes, middle stripe brown, triangular; stripes on lateral side brownish grey. Abdominal segments 7 and 8 entirely dark brown to black; hypopygium mainly dark brown, tergite 9 black. Hairs on abdomen dark brown or golden.

**Figures 55–59.**
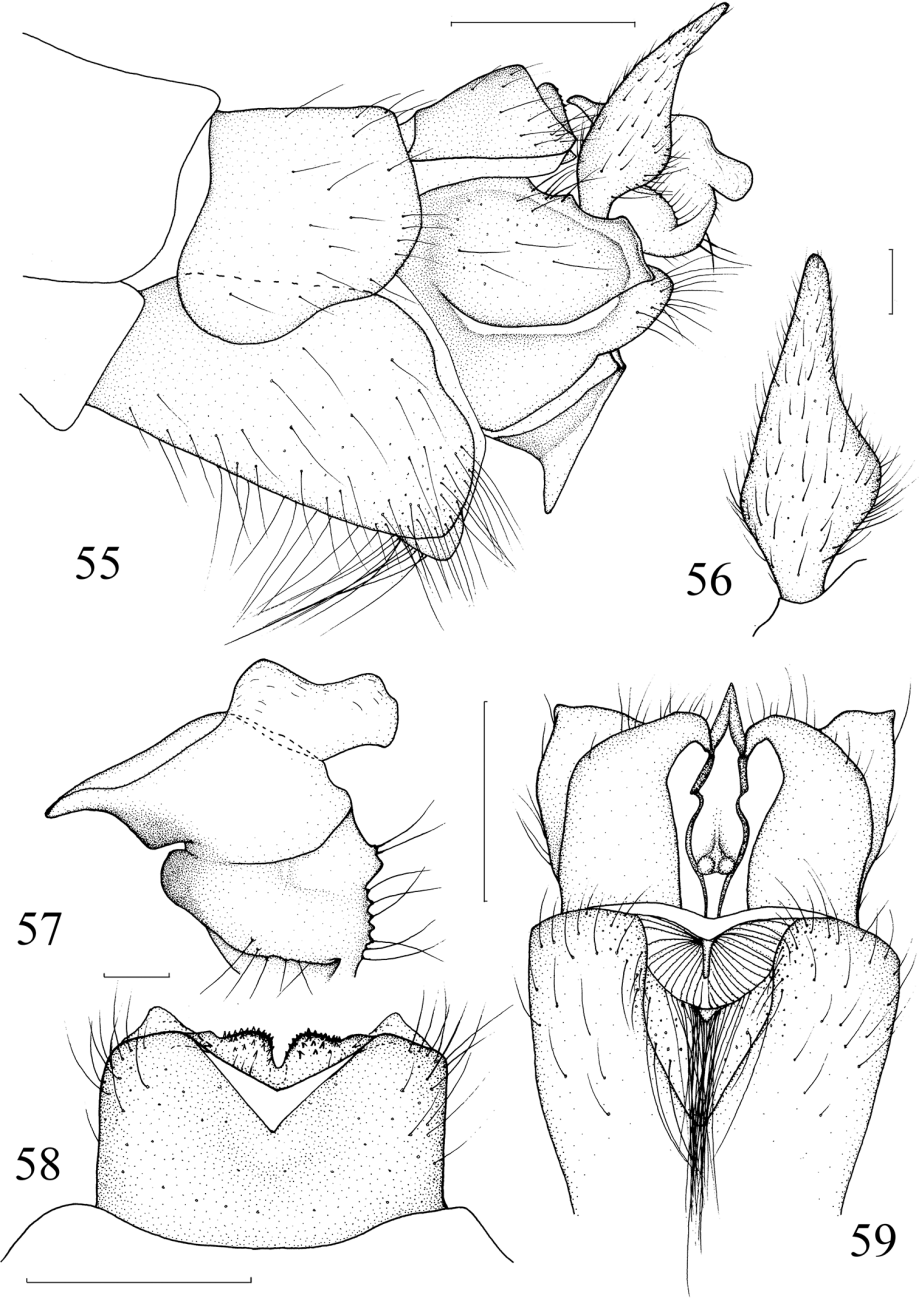
*Nephrotoma xizangensis* Yang & Yang, 1987. **55** Hypopygium, lateral view **56** outer gonostylus, lateral external view **57** inner gonostylus, lateral external view **58** tergite 9, dorsal view **59** hypopygium, ventral view. Scale bars: 0.5 mm (**55, 58, 59**); 0.1 mm (**56, 57**).

***Hypopygium*** (Figs 55–59) mainly black. Posterior extension of tergite 9 slightly depressed with two pairs of short obtuse projections; posterolateral margin with a pair of ventrad curved appendages each with two cusps (Fig. 58). Posterior margin of sternite 8 slightly depressed, middle part pleated, sclerotized, anterior part with long hairs (Fig. 55). Sternite 9 with a horn-like projection (Figs 55, 59). Outer gonostylus fleshy, widened at middle, narrowed toward tip (Fig. 56). Inner gonostylus with large concavity at base; beak sharp; outer basal lobe with hairy protuberance; posterior crest with a large membranous area (Fig. 57).

##### Distribution.

China (Xizang).

##### Remarks.

This species is similar to *N. flavonota* Alexander, 1914 from Japan (Honshu, Shikoku, Kyushu), China (Zhejiang, Fujian, Hainan), but the latter differs in the following characters: Sternite 8 sheathing very slightly narrowed outwardly, terminating in two broad lobes separated by a V-shaped emargination filled with pale membrane; lobes bearing abundant long yellow setae.

**Figures 60–62.**
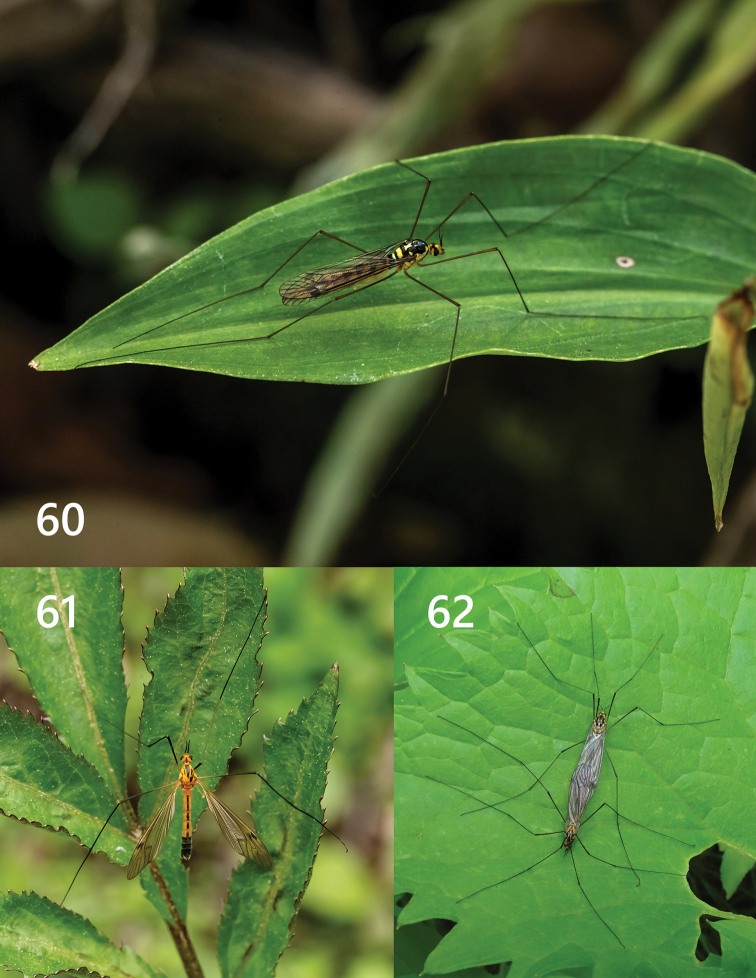
Ecograph. **60***Nephrotoma beibengensis* sp. nov. **61***Nephrotoma xizangensis* Yang & Yang, 1987 (variation) **62***Nephrotoma nigrohalterata* Edwards, 1928 (mating) (**60, 61** by Zhenqi Song **62** by Qicheng Yang).

## Supplementary Material

XML Treatment for Nephrotoma beibengensis

XML Treatment for Nephrotoma claviformis

XML Treatment for Nephrotoma didyma

XML Treatment for Nephrotoma distans

XML Treatment for Nephrotoma evittata

XML Treatment for Nephrotoma hanae

XML Treatment for Nephrotoma inorata

XML Treatment for Nephrotoma kaulbacki

XML Treatment for Nephrotoma libra

XML Treatment for Nephrotoma nigrohalterata

XML Treatment for Nephrotoma xizangensis
